# Genome-wide association study in accessions of the mini-core collection of mungbean (*Vigna radiata*) from the World Vegetable Gene Bank (Taiwan)

**DOI:** 10.1186/s12870-020-02579-x

**Published:** 2020-10-14

**Authors:** Alena Sokolkova, Marina Burlyaeva, Tatjana Valiannikova, Margarita Vishnyakova, Roland Schafleitner, Cheng-Ruei Lee, Chau-Ti Ting, Ramakrishnan Madhavan Nair, Sergey Nuzhdin, Maria Samsonova, Eric von Wettberg

**Affiliations:** 1 Peter the Great St. Petersburg Polytechnic University, Department of Applied Mathematics, St. Petersburg, Russia; 2 Federal Research Centre All-Russian N.I. Vavilov Institute of Plant Genetic Resources (VIR), St. Petersburg, Russia; 3 Kuban Branch of Federal Research Centre All-Russian N.I. Vavilov Institute of Plant Genetic Resources (VIR), Krasnodar region, Russia; 4 World Vegetable Center, Shanhua Tainan, 74199 Taiwan; 5 National Taiwan University, Taipei, 106 Taiwan; 6 World Vegetable Center, South and Central Asia, ICRISAT Campus, Patancheru, Hyderabad, Telangana 502324 India; 7 University of Southern California, Los Angeles, CA 90089 USA; 8 University of Vermont, Burlington, VT 05405 USA

**Keywords:** Mungbean, Core-collection, Phenotyping, GWAS, Population structure, Phenology traits

## Abstract

**Background:**

Mungbean (*Vigna radiata* (L.) R. Wilczek, or green gram) is important tropical and sub-tropical legume and a rich source of dietary protein and micronutrients. In this study we employ GWAS to examine the genetic basis of variation in several important traits in mungbean, using the mini-core collection established by the World Vegetable Center, which includes 296 accessions that represent the major market classes. This collection has been grown in a common field plot in southern European part of Russia in 2018.

**Results:**

We used 5041 SNPs in 293 accessions that passed strict filtering for genetic diversity, linkage disequilibrium, population structure and GWAS analysis. Polymorphisms were distributed among all chromosomes, but with variable density. Linkage disequilibrium decayed in approximately 105 kb. Four distinct subgroups were identified within 293 accessions with 70% of accessions attributed to one of the four populations. By performing GWAS on the mini-core collection we have found several loci significantly associated with two important agronomical traits. Four SNPs associated with possibility of maturation in Kuban territory of Southern Russia in 2018 were identified within a region of strong linkage which contains genes encoding zinc finger A20 and an AN1 domain stress-associated protein.

**Conclusions:**

The core collection of mungbean established by the World Vegetable Center is a valuable resource for mungbean breeding. The collection has been grown in southern European part of Russia in 2018 under incidental stresses caused by abnormally hot weather and different photoperiod. We have found several loci significantly associated with color of hypocotyl and possibility of maturation under these stressful conditions. SNPs associated with possibility of maturation localize to a region on chromosome 2 with strong linkage, in which genes encoding zinc finger A20 and AN1 domain stress associated protein (SAP) are located. Phenotyping of WorldVeg collection for maturation traits in temperate climatic locations is important as phenology remains a critical breeding target for mungbean. As demand rises for mungbean, production in temperate regions with shorter growing seasons becomes crucial to keep up with needs. Uncovering SNPs for phenology traits will speed breeding efforts.

## Background

Mungbean (*Vigna radiata* (L.) R. Wilczek, or green gram) is an important but underutilized tropical and sub-tropical legume that is a rich source of dietary protein and micronutrients and plays an vital role in restoring soil fertility in a variety of crop rotational systems. Mungbean has been of the greatest agronomic and cultural significance in India, which accounts for most of the 6 million hectares of it that are produced globally, as well as other tropical countries such as China, Indonesia, Thailand, Myanmar, and the Philippines [1]. However, it has increasing production in temperate regions such as the arid regions of Southern Europe, Queensland in Australia, the Southern Great Plains of the US, and sporadically in the Southern Russian Federation, where it can complement winter annuals such as wheat and barley due to its short duration, allowing two crop cycles per year in places where fallow seasons have previously been common. Currently mungbean accounts for about 8.5% of the world production area for grain legume crops (without counting the oilseed legume soybean) [2].

Although historically considered an orphan legume [3], considerable and significant genetic resources are accumulated in 35 genebanks of the world [4], some of which are well phenotyped. The phenotypic data of the oldest collection in the N. I. Vavilov All-Russian Institute for Genetic Resources (VIR) has been used to trace the trends of crop breeding over the past 100 years [5]. Furthermore, genomic resources have recently been developed for mungbean that allow molecular dissection of important agronomic and domestication traits. The cultivated mungbean genome was completed in 2014, complemented by a genome of its wild relatives *V. radiata* var. *sublobata* and *V. reflexo-pilosa* [6]. High-density molecular markers have been developed, allowing characterization of diversity panels such as core collections. The most widely used core-collection and mini core-collection have been developed in the World Vegetable Center (WorldVeg) [7], which has an international mandate for mungbean breeding and improvement.

Given the broad geographic range over which mungbean is produced, and the importance of phenology to allowing it to complete its lifecycle before inhospitable conditions (drought, winter cold, or the production demands of more economically valuable rotational partners such as wheat or rice), assessing the genetic basis of flowering time and related agronomic traits is critical for finding useful genetic variation for ongoing breeding efforts. Genome-wide association studies (GWAS) have emerged as powerful approach for finding genetic variation in germplasm. This approach has been used in a range of legumes, from soybean [8] and the model legume *Medicago truncatula* [9–11], to other legumes previously considered orphans such as chickpea [12, 13], cowpea [e.g., [14] and pigeonpea [15].

Here we employ GWAS to examine the genetic basis of variation in several important traits in mungbean, using the mini-core collection of mungbean established by the WorldVeg, which includes 296 accessions that represent the major market classes of mungbean and all the microsatellite alleles present in a collection of 1400 accessions [7]. This collection has been grown in common field plot in southern European part of Russia in 2018.

## Results

### Marker polymorphism analysis

**Fig. 1:**
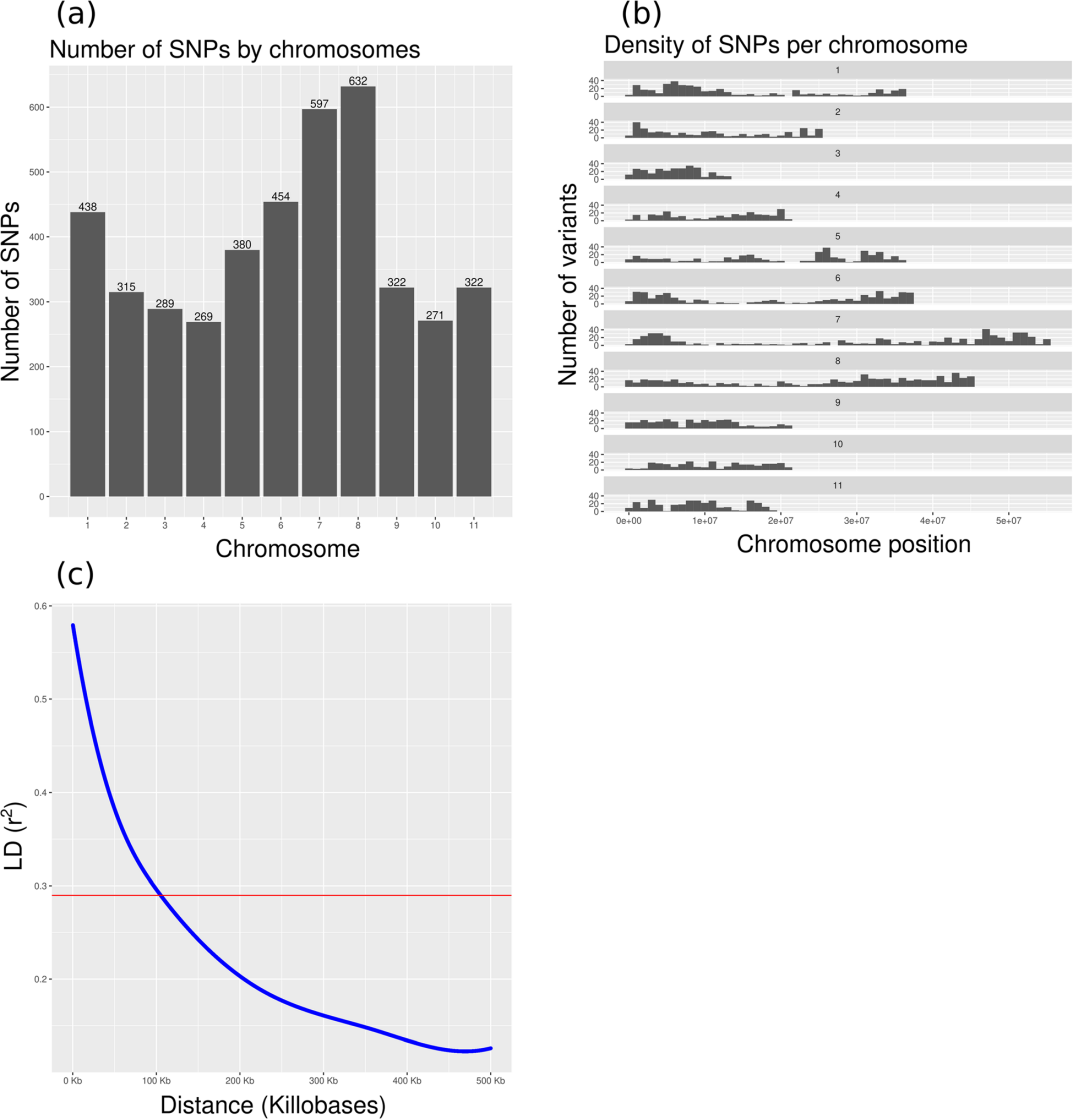
**a** Distribution of SNPs along the 11 chromosomes of the mungbean genome. **b** Density of SNPs across the mungbean genome. Chromosome 7 is the longest chromosome in the mungbean genome (55.45 Mb) and chromosome 3 is the shortest (12.93 Mb). **c** Linkage disequilibrium (r^2^) plot of mungbean. The horizontal red line indicates the half of r^2^ maximum value, which gives the critical value of r^2^

Genotyping by Sequencing (GBS) was used to survey polymorphism within the genomes of 293 accessions. SNPs were filtered to retain polymorphisms present in at least 90% of genotypes with a minor allele frequency of at least 3%. Four thousand two hundred eighty-nine polymorphisms are distributed among all chromosomes, but with variable density (from 269 SNPs on chromosome 4 to 632 SNPs on chromosome 8) and 752 SNPs are on scaffolds (Fig. 1a, b).

### Estimation of linkage disequilibrium

Linkage disequilibrium was estimated between 5041 SNPs and the half of r^2^ maximum value (r^2^ = 0.29) was taken as a critical value. The smothering spline of intra-chromosomal r^2^ values intersects this threshold at approximately 105 kb physical distance (Fig. 1c).

### Population structure of the Mungbean mini-core collection

**Fig. 2:**
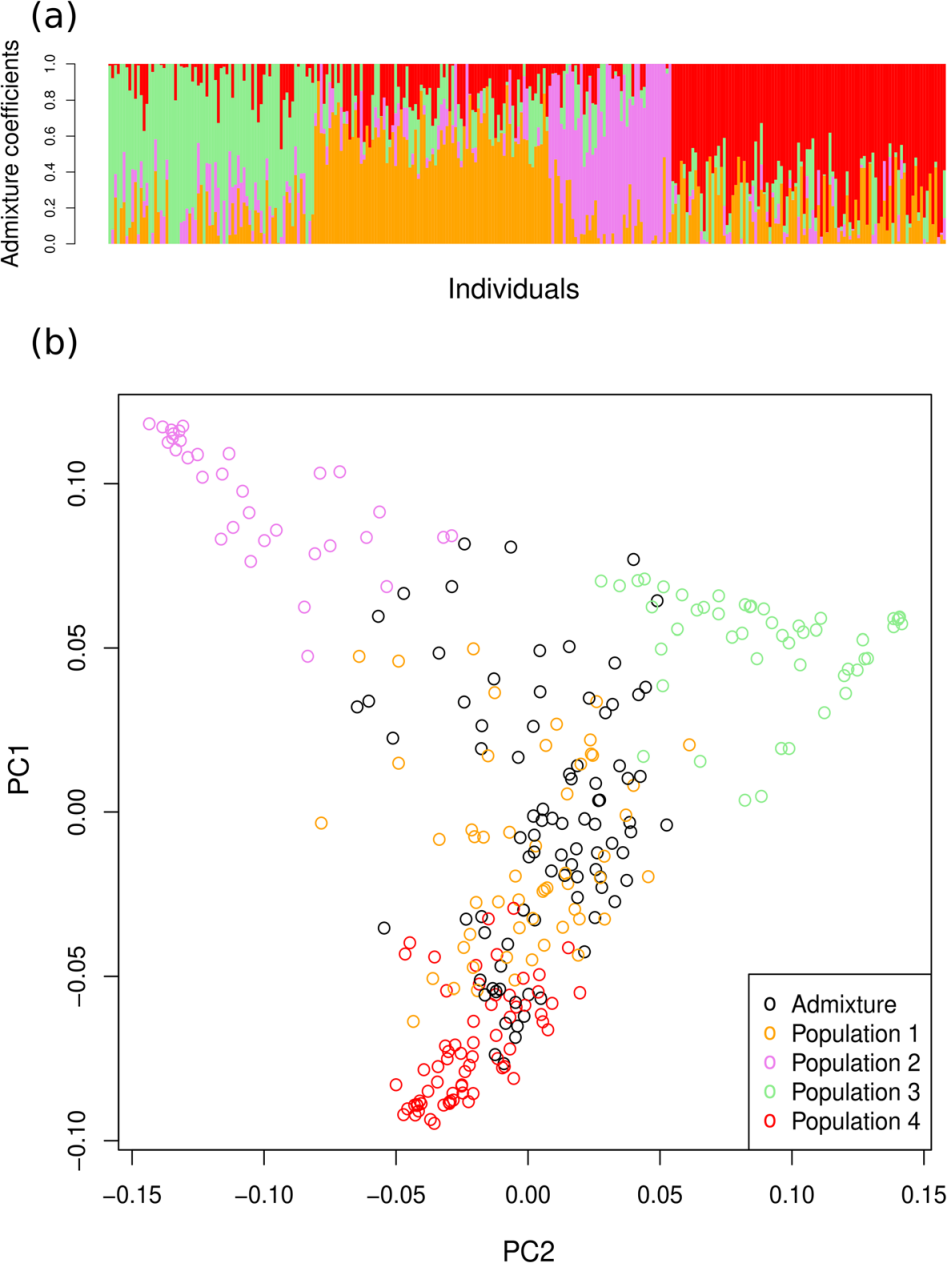
**a** Population structure of 293 mungbean accessions at K = 4. Each vertical bar represents a single accession, the length of each bar represents the proportion contributed by each population. Population 1 is color-coded orange, population 2 is color-coded violet, population 3 is color-coded green and population 4 is color-coded red. **b** Scatter plots of the first two principal components of PCA analysis based on 5041 SNPs. Each dot represents an accession color-coded according to membership (based on > 55% of identity) to populations identified from structure analysis

Structure-like Population Genetic Analysis in R package LEA [16] was used to analyze the structure of the population of the 293 accessions from the mini-core collection of mungbean and a K-value of 4 was determined to best capture the structure of the population based on minimal cross-entropy criterion (Additional file 1: Fig. S1). Using 0.55 as the likelihood to cluster for each accession in the four populations, 204 (70%) accessions were attributed to one of the four populations (Fig. 2a). The first population consists of 54 accessions, the second consists of 33 accessions, the third consists of 50 accessions and the fourth consists of 67 accessions. The remaining 89 out of 293 accessions were admixed (Additional file 2: Table S1; Additional file 3: Table S2).

Patterns of population differentiation were also analyzed using principle components (PCA). Figure 2b depicts the PCA plot for genetic data of the first versus second components and Additional file 4: Fig. S2 depicts a summary of variation and covariation attributed to the first five principle components that explained 32% of the variance of all markers.

Fixation index (F_ST_) among the four sub-populations was calculated using VCFtools [17]. Populations 1 and 4 are the most closely related (F_ST_ = 0.08) and populations 2, 3 and 4 show considerable degree of differentiation (F_ST_ = 0.42, 0.43, 0.37 respectively) (Additional file 5: Table S3).

### Single trait associations

Genetic and phenotypic data (list of phenotypes and units of measurement are in Additional file 6: Table S4) were weakly concordant, as described in Additional file 7: Table S5, which shows proportion of phenotypic variance explained by genotype for traits measured in Kuban. Neither strong positive nor negative correlation was observed between phenotypic traits (Additional file 8: Table S6).

**Fig. 3:**
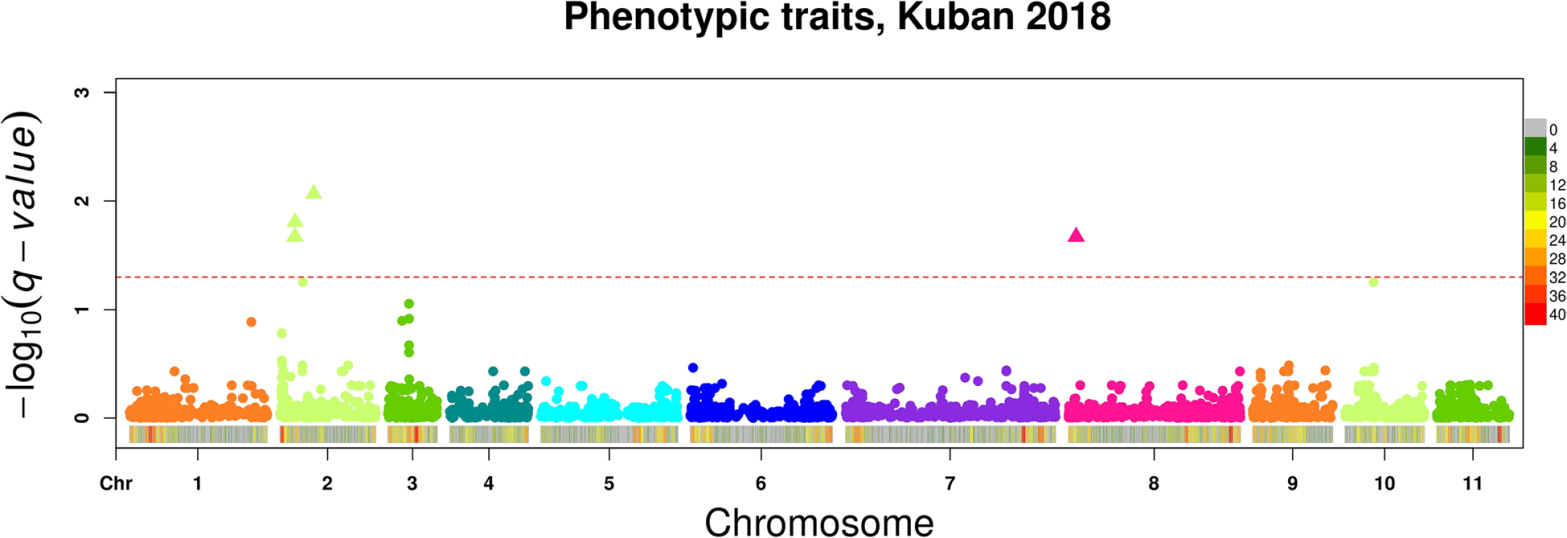
Summary of GWAS analyses for phenotype data (possibility of maturation and hypocotyl colour) measured in Kuban (different colors correspond to different chromosomes). SNPs with *q*-value < 0.05 are shown for each chromosome, marked as triangles. When one position associates with a number of phenotypes with different q-values, only the most significant SNP is represented

GWAS analysis was implemented with and without the first five PCA axes scores used as covariates for all phenotypic data. The best type of analysis was chosen for each trait separately based on genomic control parameter (λ_GC_). The analysis revealed significant associations with two phenotypic traits (possibility of maturation and hypocotyl colour), measured in Kuban (Fig. 3; Additional file 9: Fig. S3; Additional file 10: Fig. S4; Additional file 11: Table S7).

To annotate the regions in genome where significant SNPs are located, the genome was divided into haplotype blocks (haploblocks) based on linkage disequilibrium (Additional file 12: Table S8).

Four SNPs associated with possibility of maturation in Kuban territory of Southern Russia, localize to a region of 250 kb on chromosome 2 with strong linkage. Identified within this region gene Vradi02g04380 encodes zinc finger A20 and AN1 domain stress-associated protein. Stress-associated proteins (SAPs), such as A20/AN1 zinc-finger domain-containing proteins play important roles in abiotic stress signaling in plants. SAPs are important candidates for preventing the loss of yield caused by exposure to environmental stresses [18].

## Discussion

Mungbean, long an orphan legume crop of the tropical world, is emerging as a multi-faceted crop suited to a range of agroecologies. As a rich source of protein and micronutrients that can be consumed in many ways (dahl, soups, sprouts, flour, etc), and with seeds that can be rapidly cooked, it has a place in a number of traditional cuisines across Asia, and in increasingly diverse global palettes. It is a key ingredient in the new vegetarian egg (Just eggs), showing its adaptability as a protein source. Furthermore, with a short duration lifecycle, it fits into a range of crop rotations. Аdapting varieties to a range of conditions from tropical to temperate regions is necessary to meet rising global demand.

The core collection of mungbean established by the WorldVeg is a valuable resource for mungbean breeding. Two hundred ninety-three accessions that passed all filters and remained for our analysis were collected from 17 countries all over the world. 70% of accessions were attributed to one of the four populations identified in structure analysis. Populations 1 and 4 consist of accessions from Indian subcontinent (India and Pakistan), they are clustered together as seen in PCA plot (Fig. 2b). Population 2 represents accessions from Middle East and population 3 represents accessions from Southeast Asia and Oceania. In spite of the large degree of admixture, the separation of accessions into four groups on the PCA plot is quite clear (Fig. 2b). According to calculations of F_ST_ among the four sub-populations, two populations (populations 1 and 4 representing Indian subcontinent) were closely related, whereas others showed considerable degree of differentiation.

LD was estimated between 5041 SNPs and fell to half of the maximal r^2^ values at approximately 105 kb physical distance. The extent of LD reflect the effective size of population and the extent of population admixture. It is typically smaller in natural populations, strongly extended in bread cultivars, and intermediate among landraces [19].

Phenology will remain a critical breeding target for mungbean. As demand rises for mungbean, production in temperate regions with shorter growing seasons can help demand. Furthermore, with climate change, seasons in semi-arid tropical regions, such as much of South Asia and East Africa, may also become shorter. In the Kuban region in 2018 abnormally hot weather persisted through the flowering and beginning of maturation phases of some accessions and the development of the accessions was extremely non-uniform. This non-uniformity was elicited by plant exposure to different photoperiod in conjunction with drought, and represents plant response to these incidental stresses.

By performing GWAS on mungbean mini-core collection planted in Southern Russia, we have found several loci significantly associated with maturation and colour of hypocotyl. We found that SNPs associated with possibility of maturation in Kuban localize to a region on chromosome 2 with strong linkage in which genes encoding zinc finger A20 and AN1 domain SAP are located. SAPs are known to prevent the loss of yield caused by environmental stresses [18]. Uncovering SNPs for these traits will speed breeding efforts for production of mungbean suitable for cultivation in temperate regions. Furthermore, understanding correlations phenology with other traits such as seed size or leaf shape, will be necessary to adapt new varieties to changing agroecological conditions.

## Conclusions

Here we perform GWAS in accessions of mungbean, using the mini-core collection established by the WorldVeg. The collection has been grown in southern European part of Russia in 2018 under incidental stresses caused by abnormally hot weather and different photoperiod. We have found several loci significantly associated with maturation and colour of hypocotyl. We found that SNPs associated with possibility of maturation in Kuban localize to a region on chromosome 2 with strong linkage, in which genes encoding zinc finger A20 and AN1 domain SAP are located. SAPs are known to prevent the loss of yield caused by environmental stresses. Phenotyping of this collection for maturation traits in temperate climatic locations is important for future breeding applications.

## Methods

### Plant materials

The mungbean (*Vigna radiata*) minicore collection was assembled from accessions held in trust under the multilateral system by the World Vegetable Center genebank as described in [7]. Seed of this collection was received from the World Vegetable Center with a Standard Material Transfer Agreement. Seed of the collection is available to the public (http://seed.worldveg.org/) (see Additional file 2: Table S1).

### Phenotyping

Phenotyping of 293 accessions was performed at Kuban experimental station of VIR in 2018.

Kuban experimental station of VIR is located at 45°22΄ N, 40°37΄E in the steppe zone on the Prikubanskaya plain, 80 km from the beginning of the spurs of the Caucasian ridge. The soil is a weakly leached chernozem. The humus content in the surface horizons of the soil is 3.6–4.6%.

The climate near Kuban is temperate continental, with hot summers characterized by insufficient moisture and by extreme instability of all climatic elements. The average monthly temperature of the coldest month, January, is 2.6 °C, and the warmest, July, 23 °C. The average annual rainfall is 565 mm.

Sowing was carried out on May the 5th. The summer had insufficient rainfall. The average air temperature was 24.4 °C, which is 2.2 °C higher than normal. The absolute maximum of summer air temperature of 39.9 °C was observed in June - during the period of juvenile plant growth. In June, high air temperatures and lack of precipitation led to soil and atmospheric drought. Abnormally hot weather persisted throughout July - during flowering and the beginning of maturation of some accessions. Twenty-eight days with dry winds were noted, of which 18 days in August. Precipitation during the summer was 73 mm, which is 40% of the norm.

The development of the accessions was extremely non-uniform. The first selective harvest of beans to avoid their cracking began in the second decade of July. The last accessions have been harvested at the first decade of October in 116 days after sawing, when night temperatures dropped to 0.3 °C, which is below the optimum for the development of mungbean plants. One hundred twenty-eight accessions did not enter the ripening stage.

The following traits were recorded in the field: hypocotyl color, plant habit, days to 50% flowering, days to first mature pods, days to maturity, plant height, number of pods per plant, pod length, number of seeds per pod, 100 seed weight, plant weight and yield. Only 35 samples matured, thus we took only six traits for further analysis and transformed days to maturity to categorical trait possibility of maturation (whether the sample matured or not). The list of phenotypes and units of measurement are in Additional file 6: Table S4.

### Phenotyping data analyses

Shapiro-Wilk test for normality [20] was implemented to quantative phenotypic traits. Spearman correlation coefficients of traits were calculated using the “rcorr” function from the “Hmisc” R library [21].

### Genotyping by sequencing (GBS) and SNP calling

Total genomic DNA was extracted from bulked young leaves of three single plants. GBS was performed at Diversity Array Technology P/L (DArT). The sequence data generated were then aligned to the mungbean reference genome sequence, Vradi_ver6 [6] to identify single nucleotide polymorphisms (SNPs).

SNPs were further filtered using VCFtools [17] to require minor allele frequency (MAF) > 3% and genotype call-rate > 90%, hence 5041 SNPs in 293 accessions passed all filters remained for further analysis.

### Genetic data analyses

Principal component analysis (PCA) was conducted using the “SNPRelate” R library [22]. Custom scripts in R [23] were used to plot depth and distribution of SNPs on chromosomes.

### Estimation of linkage disequilibrium (LD)

LD was estimated using the squared correlation coefficient (r^2^) between genotypes. VCFtools [17] was used to calculate intra-chromosomal r^2^ values. Only r^2^ values for SNPs with the physical distance between markers less than 500 kb were used to plot LD decay graph. LD decay was assessed by fitting a smooth spline of intra-chromosomal r^2^ values against the physical distance (bp) between markers in R [23]. The half of r^2^ maximum value was taken as a critical value. The threshold beyond which the LD was accepted as real physical linkage was estimated to be r^2^ = 0.29. The intersection of the smothering spline of intra-chromosomal r^2^ values with this threshold was considered to be an estimate of the range of LD.

### Estimation of the proportion of phenotypic variance explained by all genome-wide SNPs

The GCTA program [24] was used to estimate the proportion of variance in phenotypes explained by all genome-wide SNPs. First, phenotypic data were normalized. Then, the genetic relationships among individuals from genome-wide SNPs were calculated using GCTA-GRM analysis. And, finally, GCTA-GREML analysis was performed to estimate the proportion of variance in a phenotype explained by all GWAS SNPs (i.e. the SNP-based heritability).

### Analysis of population structure

Structure-like Population Genetic Analysis in R package LEA [16] was used to analyze the structure of the population of the 293 accessions from the mini-core collection of mungbean. Choosing the number of subpopulations is based on a cross-entropy criterion. This criterion is based on the prediction of a fraction of masked genotypes (matrix completion), and on the cross-validation approach. Smaller values of the cross-entropy criterion usually mean better runs. Ten independent runs were performed for each simulated value of K, ranging from 1 to 10. K-value for which the cross-entropy curve exhibits a plateau was chosen (K = 4). An individual accession with more than 55% identity from a single sub-population was classified as representative of that sub-population. Fixation index (F_ST_) among the four sub-populations was calculated using VCFtools [17].

### Mapping approaches

GWAS analysis was performed using a single-locus linear mixed model, implemented in FaST-LMM toolset (Factored Spectrally Transformed Linear Mixed Models) [25]. Principal component analysis (PCA) of 5041 SNPs revealed that the first five significant principal components (PCs) explained 32% of the variance of all markers. The LMM model was implemented with and without the first five PCA axes scores used as covariates for all phenotypic data. The best type of analysis was chosen for each trait separately based on genomic control parameter (λ_GC_). We used a false discovery rate (FDR) [26] of 0.05 to determine significant trait associated loci separately for each trait. Manhattan plots were constructed using “CMplot” library [27] in R. Annotation of significant associated markers was performed using the Legume information system (LIS) [28] database.

### Estimation of haploblocks

To divide the genome into haplotype blocks (haploblocks) based on linkage disequilibrium, Haploview tools [29] were applied to the set of 4289 SNPs, located on chromosomes. Chromosomal regions with strong linkage were identified using default Haploview parameters (confidence interval for LD [0.7, 0.98]). Each haploblock was considered as the set of SNPs located within a given haploblock and used for annotation of significant associated markers.

## Supplementary information

**Additional file 1: Figure S1.** Cross-entropy plot for 293 mungbean accessions. X-axis indicates the number of ancestral populations, Y-axis represents the minimal cross-entropy. Ten independent runs were performed for each simulated value of K, ranging from 1 to 10. K-value for which the cross-entropy curve exhibits a plateau was chosen (K = 4).

**Additional file 2: Table S1.** List of accessions, collection sites and accession’s groups. Four populations were identified using Structure-like Population Genetic Analysis in R package LEA. An individual accession with more than 55% identity from a single sub-population was classified as representative of that sub-population. The remaining accessions were considered as admixture.

**Additional file 3: Table S2.** Number of mungbean accessions in populations among the 17 countries of origin.

**Additional file 4: Figure S2.** Scatter plots of the first five principal components of PCA analysis based on 5041 SNPs. Each dot represents an accession. Color-coded is according to membership (based on > 55% of identity) to populations identified from structure analysis.

**Additional file 5: Table S3.** Fixation index (F_ST_) among the four sub-populations.

**Additional file 6: Table S4.** List of phenotypes measured at Kuban in 2018.

**Additional file 7: Table S5.** The proportion of variance in a phenotype explained by all GWAS SNPs (i.e. the SNP-based heritability) for traits measured in Kuban.

**Additional file 8: Table S6.** The correlation coefficients between phenotypic traits.

**Additional file 9: Figure S3.** Summary of GWAS analyses for phenotypic trait Possibility of maturation measured in Kuban. (a) SNP QQ-plot. (b) SNP Manhattan plot (different colors correspond to different chromosomes). SNPs with q-value < 0.05 are shown for each chromosome, marked as triangles.

**Additional file 10: Figure S4.** Summary of GWAS analyses for phenotypic trait Hypocotyl color measured in Kuban. (a) SNP QQ-plot. (b) SNP Manhattan plot (different colors correspond to different chromosomes). SNPs with q-value < 0.05 are shown for each chromosome, marked as triangles.

**Additional file 11: Table S7.** Significant SNPs for phenotypic traits measured in Kuban. Results of GWAS analysis for phenotypic traits measured in Kuban, 2018. Annotation of significant associated markers was performed using Legume information system (LIS) database.

**Additional file 12: Table S8.** Haploblocks inferred by Haploview tools.

## Data Availability

The sequence data are available from the National Center for Biotechnology database under BioProject PRJNA645721.

## Abbreviations

GWAS: Genome-wide association studies
SAP: Stress associated protein
WorldVeg: World Vegetable Center
SNP: Single nucleotide polymorphism
VIR: N. I. Vavilov All-Russian Institute for Genetic Resources
GBS: Genotyping by Sequencing
PCA: Principal component analysis
F_ST_: Fixation index
LD: Linkage Disequilibrium
DarT: Diversity Array Technology P/L
MAF: Minor allele frequency
FaST-LMM: Factored Spectrally Transformed Linear Mixed Models
FDR: False discovery rate
