# Supplementary figures and images for: Genome-wide association study in accessions of the mini-core collection of mungbean (Vigna radiata) from the World Vegetable Gene Bank (Taiwan)
Source: BMC Plant Biol. 2020 Oct 14;20(Suppl 1):363. doi: 10.1186/s12870-020-02579-x (PMC7556912; doi:10.1186/s12870-020-02579-x)

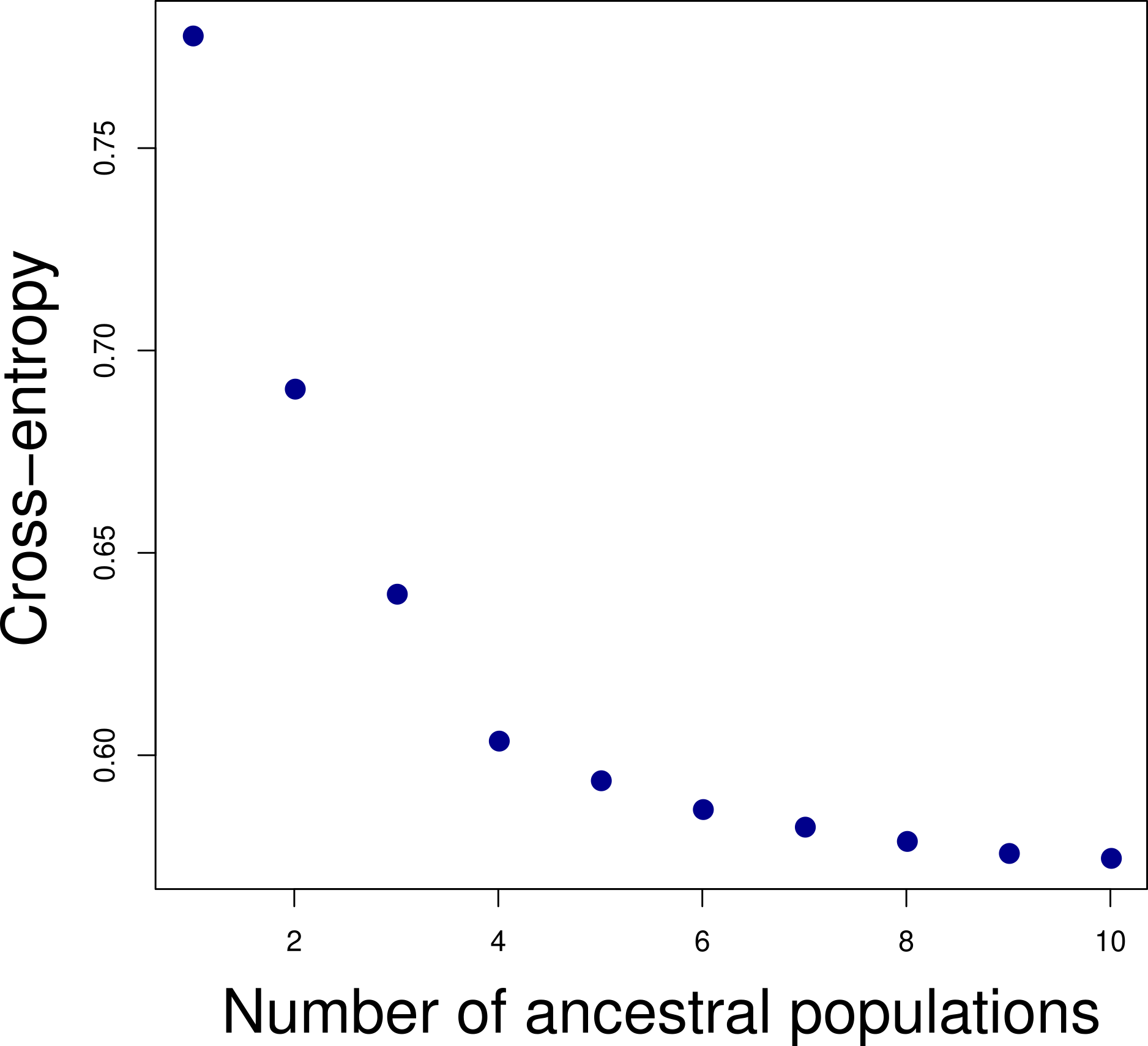

Supplement: Supplementary file 1 — Additional file 1: Figure S1. Cross-entropy plot for 293 mungbean accessions. X-axis indicates the number of ancestral populations, Y-axis represents the minimal cross-entropy. Ten independent runs were performed for each simulated value of K, ranging from 1 to 10. K-value for which the cross-entropy curve exhibits a plateau was chosen (K = 4). [file 12870_2020_2579_MOESM1_ESM.png]

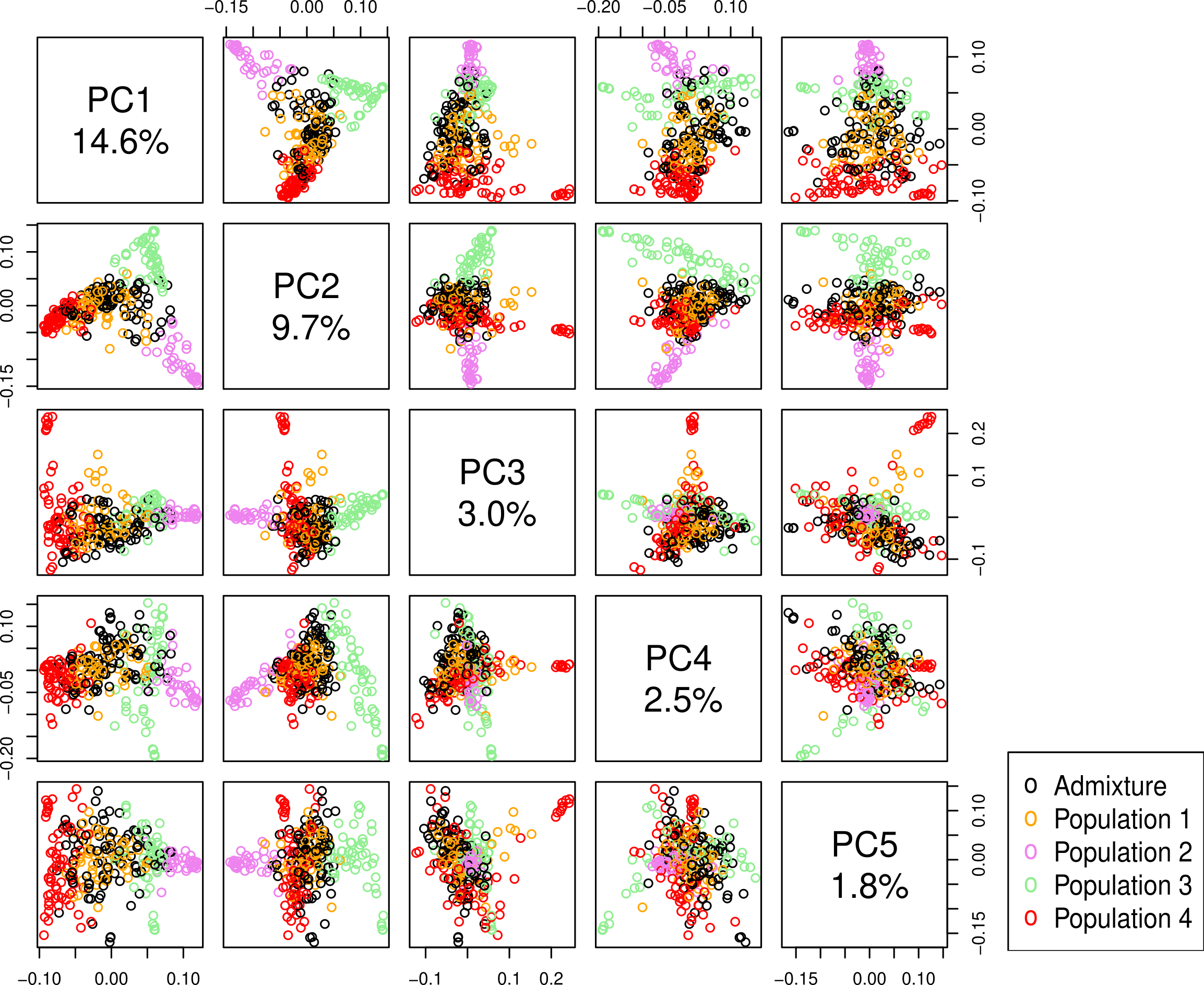

Supplement: Supplementary file 4 — Additional file 4: Figure S2. Scatter plots of the first five principal components of PCA analysis based on 5041 SNPs. Each dot represents an accession. Color-coded is according to membership (based on > 55% of identity) to populations identified from structure analysis. [file 12870_2020_2579_MOESM4_ESM.png]
