# Supplementary material for: Genome-wide association study in accessions of the mini-core collection of mungbean (Vigna radiata) from the World Vegetable Gene Bank (Taiwan)
Source: BMC Plant Biol. 2020 Oct 14;20(Suppl 1):363. doi: 10.1186/s12870-020-02579-x (PMC7556912; doi:10.1186/s12870-020-02579-x)
Supplement: Supplementary file 5 — Additional file 5: Table S3. Fixation index (FST) among the four sub-populations. [file 12870_2020_2579_MOESM5_ESM.docx]

**Table S3 Fixation index (F_ST_) among the four sub-populations.**

|  | Population 2 | Population 3 | Population 4 |
| --- | --- | --- | --- |
| Population 1 | 0.26 | 0.17 | 0.08 |
| Population 2 |  | 0.42 | 0.43 |
| Population 3 | 0.42 |  | 0.37 |
