# Supplementary material for: Genome-wide association study in accessions of the mini-core collection of mungbean (Vigna radiata) from the World Vegetable Gene Bank (Taiwan)
Source: BMC Plant Biol. 2020 Oct 14;20(Suppl 1):363. doi: 10.1186/s12870-020-02579-x (PMC7556912; doi:10.1186/s12870-020-02579-x)
Supplement: Supplementary file 6 — Additional file 6: Table S4. List of phenotypes measured at Kuban in 2018. [file 12870_2020_2579_MOESM6_ESM.docx]

**Table S4 List of phenotypes measured at Kuban in 2018.**

| **Phenotype** |
| --- |
| Hypocotyl colour, score |
| Plant habit, score |
| Days to 50% flowering, days |
| Days to first mature pods, days |
| Possibility of maturation, score |
| Plant height, cm |
