# Supplementary material for: Genome-wide association study in accessions of the mini-core collection of mungbean (Vigna radiata) from the World Vegetable Gene Bank (Taiwan)
Source: BMC Plant Biol. 2020 Oct 14;20(Suppl 1):363. doi: 10.1186/s12870-020-02579-x (PMC7556912; doi:10.1186/s12870-020-02579-x)
Supplement: Supplementary file 7 — Additional file 7: Table S5. The proportion of variance in a phenotype explained by all GWAS SNPs (i.e. the SNP-based heritability) for traits measured in Kuban. [file 12870_2020_2579_MOESM7_ESM.docx]

**Table S5 The proportion of variance in a phenotype explained by all GWAS SNPs (i.e. the SNP-based heritability) for traits measured in Kuban ***.

| **Phenotype** | **Genotype variation (SE**)** | **Phenotype variation (SE**)** | **SNP-based heritability (SE**)** | **p-value** |
| --- | --- | --- | --- | --- |
| Days to 50% flowering | 0.011696  (0.004382) | 0.052827  (0.004873) | 0.221397  (0.073646) | 2.683e-09 |
| Possibility of maturation | 0.009530  (0.006273) | 0.105317  (0.008892) | 0.090492  (0.058033) | 0.006992 |
| Hypocotyl color | 0.037426  (0.013846) | 0.161058  (0.014612) | 0.232376  (0.075568) | 1.491e-07 |
| Plant height | 0.003266  (0.002150) | 0.022897  (0.002387) | 0.142657  (0.089925) | 0.03536 |

* - Only statistically significant measurements are represented.

** - Standard Error.
