# Supplementary material for: Genome-wide association study in accessions of the mini-core collection of mungbean (Vigna radiata) from the World Vegetable Gene Bank (Taiwan)
Source: BMC Plant Biol. 2020 Oct 14;20(Suppl 1):363. doi: 10.1186/s12870-020-02579-x (PMC7556912; doi:10.1186/s12870-020-02579-x)
Supplement: Supplementary file 8 — Additional file 8: Table S6. The correlation coefficients between phenotypic traits. [file 12870_2020_2579_MOESM8_ESM.docx]

**Table S6 The correlation coefficients between phenotypic traits ***.

|  | Days to 50% flowering | Days to first mature pods | Plant habit |
| --- | --- | --- | --- |
| Days to first mature pods | 0.47 |  |  |
| Hypocotyl color | -0.17 |  |  |
| Plant habit |  | -0.18 |  |
| Plant height | 0.27 |  | -0.35 |

* - Only statistically significant measurements are represented.
